# Sustainable Solar Evaporation from Solute Surface via Energy Downconversion

**DOI:** 10.1002/gch2.202000077

**Published:** 2020-10-26

**Authors:** Yue Bian, Yanli Tian, Kun Tang, Wei Li, Lijuan Zhao, Yi Yang, Jiandong Ye, Shulin Gu

**Affiliations:** ^1^ School of Electronic Science and Engineering Nanjing University Nanjing 210093 China; ^2^ School of the Environment Nanjing University Nanjing 210093 China

**Keywords:** solar vapor, solute recovery, waste water, water purification

## Abstract

Solar‐powered interfacial evaporation, a cost‐effective and ecofriendly way to obtain freshwater from contaminated water, provides a promising path to ease the global water crisis. However, solute accumulation has severely impacted efficient light‐to‐heat‐to‐vapor generation in conventional solar evaporators. Here, it is demonstrated that an interfacial solar thermal photo‐vapor generator is an efficient light‐to‐heat photo‐vapor generator that can evaporate water stably in the presence of solute accumulation. An energy downconversion strategy which shifts sunlight energy from visible‐near infrared to mid infrared‐far infrared bands turns water from transparent to its own absorber, thus changing the fixed evaporation surface (black absorber) in a traditional solar evaporator to a dynamic front (solute surface). Light reflected from the solute can be recycled to drive evaporation. The prototype evaporator can evaporate at a high speed of 1.94 kg m^−2^ h^−1^ during a persistent solute accumulation process for 32 h. Such an ability to produce purified water while recycle valuable heavy metals from waste water containing heavy metal ions can inspire more advanced solar‐driven water treatment devices.

## Graphical abstract

An interfacial solar thermal photo‐vapor generator which shifts sunlight energy from visible‐near infrared to mid infrared‐far infrared bands can enable sustainable and efficient water evaporation from a solute surface as water serves as its own absorber at the dynamic evaporation front (solute layer) and the reflected light can be recycled.

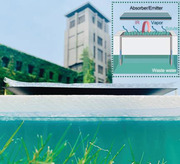

## 1 Introduction

Access to clean, reliable, and affordable freshwater is among the largest global challenges of the 21st century.^[^ ^1^ ^]^ Global population growth, climate change, economic development, and competition over finite, vulnerable, water resources are exerting growing pressure on our earth in spite of human's unquenchable thirst for water.^[^ ^2^ ^]^ Solar‐driven water evaporation, a simple and effective way to produce safe and drinkable freshwater from saline water, domestic and industrial wastewater in the absence of energy infrastructures, is rising as a promising sustainable solution to alleviate such pressure from the supply side.^[^ ^3^ ^]^

Recently, the advanced interfacial solar‐thermal vapor generation systems (iSTV) are well designed with typical three layers—solar absorbing layer, water supplying layer, and thermal insulating layer—in terms of function, floating on the water surface and generating vapor efficiently.^[^ ^4^ ^]^ Among them, carbonization of very low cost biomass source materials such as woods,^[^ ^4i^ ^]^ magnolia fruits,^[^ ^4k^ ^]^ loofah,^[^ ^4l^ ^]^ pencil waste,^[^ ^4m^ ^]^ just to name a few, are promising for fabricating scalable and economical evaporators. However, solute accumulation has severely told on efficient light‐to‐heat‐to‐vapor generation in conventional solar evaporators due to the interruption of light absorption and vapor escape channels by sediments.^[^ ^5^ ^]^ All sustainability aims were thus centered on avoiding solute accumulation on the evaporation surface. A suite of pioneering strategies such as “hydrophobic design,”^[^ ^6^ ^]^ “Janus design,”^[^ ^7^ ^]^ “contactless design,”^[^ ^8^ ^]^ “reflow design,”^[^ ^9^ ^]^ “sidewall precipitation,”^[^ ^10^ ^]^ “edge‐preferential crystallization,”^[^ ^11^ ^]^ and “localized crystallization”^[^ ^12^ ^]^ have been reported as viable solutions for sustainable evaporation. Recently, Tan and co‐workers systematically investigated the currently available salt‐rejection strategies for long‐term desalination.^[^ ^5a^ ^]^ Li et al. reviewed the advanced structural designs and engineering strategies for salt removal.^[^ ^5b^ ^]^ It is worth to note that Tan's group recently reported a novel “fluidic design” which is able to guarantee complete salt rejection. In most cases, solar evaporators can hardly achieve high conversion efficiency (evaporation rate) while having a good salt rejection capability, in this paper, the authors have done a nice job achieving them simultaneously.^[^ ^13^ ^]^ Still, it is worthwhile to reimagine whether we can achieve sustainable evaporation without avoiding surface accumulation.

In this work, we show an interfacial solar thermal photo‐vapor generator (iSTPV) can evaporate at a high speed of 1.94 kg m^−2^ h^−1^ during a persistent solute accumulation process for 32 h when treating wastewater. Different from the solar‐to‐thermal‐to‐vapor process in conventional iSTV, current iSTPV undergos a solar‐to‐thermal‐to‐photo‐to‐vapor process by downconverting the sunlight energy from visible‐near infrared (VIS‐NIR) to mid infrared (MIR)‐far infrared (FIR) bands where water absorbs strongly. The advantage of this conversion is to change the fixed evaporation surface (black absorber) which is easily affected by solute in traditional solar evaporator into a dynamic evaporation front (solute surface). Moreover, light reflection from the solute can be recycled to drive evaporation in iSTPV. In the following section, we will describe in detail how the device was manufactured and show its performance in the laboratory and outdoors.

## 2 Results and Discussion

**Figure 1:**
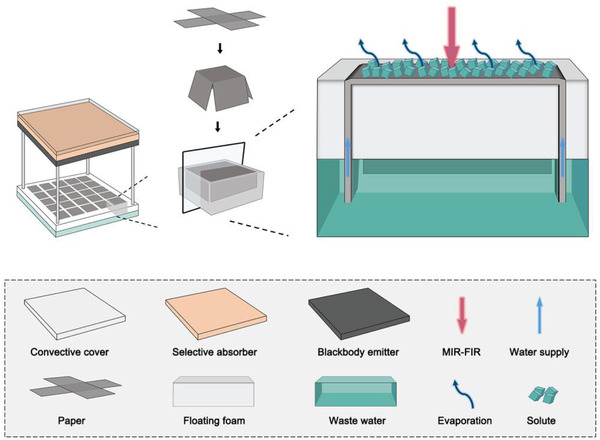
Schematic illustrations of the interfacial solar thermal photo vapor‐generator based on energy downconversion design. Such design can enable stable vapor generation and solute recovery among which the selective absorber/blackbody emitter shifts the sunlight energy from UV–VIS–NIR to MIR‐FIR band where water act as its own absorber efficiently, the paper is responsible for 2D water supply, and the floating foam provides mechanical support while ensuing heat localization. With the continuous escape of water, solutes accumulate on the surface of paper and sucks water from the underlying paper layer for further evaporation.

**Figure** **1** shows the schematic illustrations of the iSTPV. The solar selective absorber (SSA) covered with a transparent convective cover is used to capture sunlight effectively while minimize the upward thermal loss caused by convection and radiation. In the meantime, the blackbody emitter reradiates the captured solar energy in the form of infrared photons whose wavelength are above 2 µm which are then directly absorbed by a very thin layer of water. The water layer sub‐100 µm can readily absorb >99% of the infrared photons via vibrate absorption.^[^ ^14^ ^]^ The paper layer is used to draw a thin layer of water from the bulk water, and the floating foam provides mechanical support while ensuring heat localization.^[^ ^4b^ ^]^ With the continuous escape of water, solutes accumulate on the surface of paper and suck water from the underlying paper layer for further evaporation.

**Figure 2:**
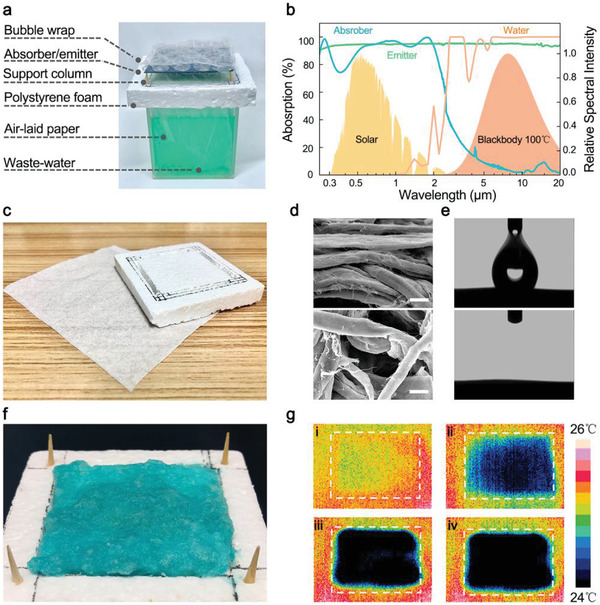
Fabrication and characterization of an iSTPV. a) Photograph of the as‐prepared lab‐scale iSTPV. b) Absorption curves of the selective absorber and blackbody emitter, and calculated absorptance of a 100 µm thick water layer in UV–VIS–NIR–MIR band. c) Optical image of the low‐cost polystyrene foam and air‐laid paper used for the construction of 2D water supply channel. d) The side‐view (upper) and top‐view (lower) SEM images of the air‐laid paper. Scale bar, 10 µm. e) Water (100 µL) permeates through the paper rapidly (240 ms). f) Photograph of the paper showing the solute accumulation after water evaporation. g) IR images of the dry solute/paper i) before and ii, iii, iv) after 2, 108, and 144 s touching with water.

**Figure** **2**a shows a picture of the as‐prepared lab‐scale iSTPV with an evaporation area of 45 mm × 45 mm. Details of the manufacturing process can be find in Figure S1 in the Supporting Information. As shown in Figure 2b, the SSA is designed as a highly efficient absorber in the solar spectrum range (absorbance: 0.92, weighted by the AM 1.5G solar spectrum) and poor absorber in the MIR range (emittance: 0.04, weighted by the 100 °C blackbody emitter). Solar energy captured was then radiated by the black emitter (emittance: 94.7%). The light absorption property of a 100 µm thick water layer was also provided (Figure 2b and Figure S2, Supporting Information). It is intuitive that a thin layer of water hardly absorbs photons in the solar spectrum while over 99% photons can be absorbed in the MIR range.

Appropriate thickness of water and rapid water transport are key factors for efficient and sustainable solar evaporation. We now know that 100 µm thick water layer is fine for efficient evaporation, because too thin water layer cannot achieve effective photon absorption, and when water is too thick, heat is wasted on heating unnecessary water instead of promoting evaporation. So the question is, how can we create a water layer 100 µm thick. Our solution is to stack two sheets of air‐laid paper about 50 µm thick (Figure 2c). Figure 2d shows the scanning electron microscope (SEM) images of the air‐laid paper. It can be seen from the side‐view image that flat cellulose fibers are randomly stacked together to form a paper layer about 50 µm thick. The loosely packed fibers created numerous micropores (diameter: ≈10–50 µm) among the paper layer. These pores help to the formation of capillary pressure and the rapid transport of water.^[^ ^15^ ^]^ It is also worth to note that the hydrophilic nature of the cellulose fibers also contributes to effective water absorption. As can be seen from Figure 2e, a drop of water can permeate through the paper in 240 ms. With the continuous escape of water, solutes accumulate on the surface of paper (Figure 2f). Under the synergistic help of the hydrophilic nature of cellulose fibers and the capillary force, water is continuously and rapidly transported to the evaporation front, even through the solute layer, enabling sustainable evaporation. The wetting process of the dry solute/paper after touching with water was visualized by an infrared camera. Only ≈144 s is needed for the solute/paper to wick water from the bottom bulk water to its upper surface.

**Figure 3:**
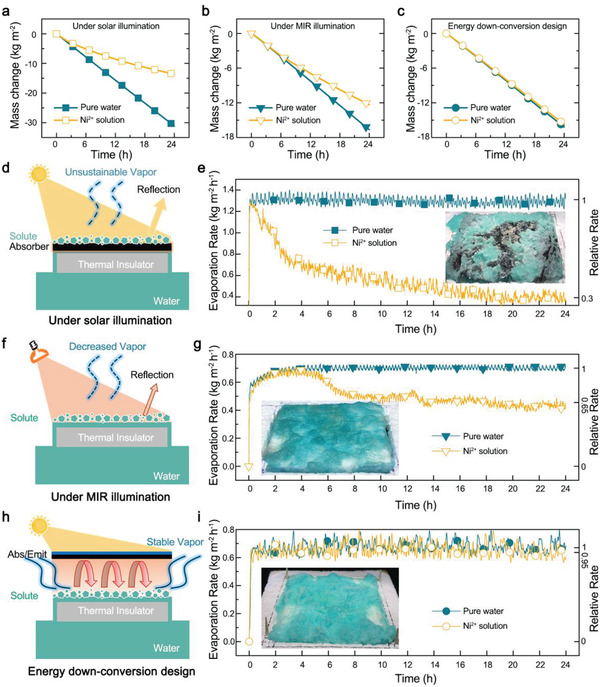
Evaporation performance during solute accumulation. a–c,e,g,i) Mass changes of a–c) water and e,g,i) corresponding water evaporation rates using a,e) a typical conventional iSTV under 1‐sun illumination, b,g) the iSTPV without the absorber/emitter structure under MIR illumination, and c,i) the iSTPV under 1‐sun illumination, that is the energy downconversion design. Insets in (e), (g) , and (i), photos of the paper surface after 24 h of continuous illumination. d) Schematic shows the accumulated solutes reflect sunlight strongly, resulting in unsustainable vapor generation in conventional iSTV. f) Schematic illustrating the low reflection of solutes lead to decreased while sustainable vapor generation under direct MIR illumination where water acts as its own absorber. h) Schematic illustrating the working principle of iSTPV as an efficient and stable solar evaporator during solute accumulation.

Next, the evaporation performance during solute accumulation was evaluated carefully. Saturated Ni^2+^ solution is used as a representative of industrial wastewater. The long‐term stability of conventional iSTV with 2D water supply channel was evaluated at first. Here, plasmonic TiN nanoparticles with the diameter of 40–50 nm was selected as a representative black absorber because of its high internal photothermal conversion efficiency (96.7%) and spray‐coated on the air‐laid paper.^[^ ^16^ ^]^ The mass changes of water using the hybrid plasmonic TiN/paper iSTV under 1‐sun illumination were recorded (**Figure** **3**a). The TiN based iSTV evaporates pure water at a high rate of 1.31 kg m^−2^ h^−1^, which is × 2.7 that of bare Ni^2+^ solution under 1‐sun illumination (Figure S4, Supporting Information). Over 31 kg m^−2^ water was evaporated after 24 h of illumination. However, the mass change decreased to 14 kg m^−2^ when treating Ni^2+^ solution. The reason is that solute deposits rapidly on the surface of the absorber, hinders light absorption and vapor escape (Figure 3d), which is evident from the evolution of evaporation rates (Figure 3e). Note that, the evaporation rate decreased to 30% of the original value after 24 h illumination, which means that the 2.7‐fold evaporation advantage mentioned above is no longer available for wastewater treatment.

What if we change the solar light to mid infrared light? The absorber/emitter layer in iSTPV was thus removed, letting the air‐laid paper layer irradiated directly under an MIR light source. Interestingly, the difference in the total amount of evaporated water when treating pure water and wastewater decreased compared to working under sunlight (Figure 3b). The reason is that different from illumination under solar light, water was pumped to the upper surface of solute and evaporates continuously as it serves as its own absorber under MIR band, despite some light was reflected by the accumulated solute (Figure 3f), which is also evident from the evolution of evaporation rates (Figure 3g). As can be seen from Figure 3g, when treating wastewater under MIR illumination, the evaporation rate shows little difference with that of treating pure water in the first 4 h and gradually decreased to a stable value, corresponding to the process in which the air‐laid paper is gradually covered with solute.

Next, the absorber/emitter layer is returned to iSTPV and illuminated by sunlight, that is the energy downconversion design. It is interesting to see that the mass changes of water shows little difference when treating pure water and wastewater (Figure 3c). The reason is that light reflection from the solute layer is recycled by the black emitter which in turn promotes further evaporation. This process is quite similar to the greenhouse effect that provides the warmth of our ecosystem, in which the infrared radiation from the earth's surface, that should have dissipated to the outer atmosphere to balance the earth's temperature, is absorbed and re‐radiated back by the increasing greenhouse gasses.^[^ ^17^ ^]^ By leveraging this artificial greenhouse effect in iSTPV, a sustainable evaporation rate was achieved when solute accumulation (Figure 3i).

**Figure 4:**
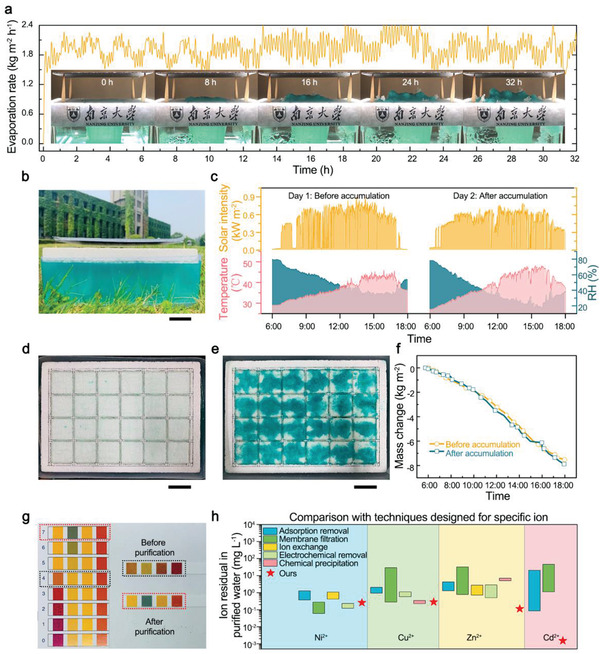
iSTPV enabled water purification and solute recovery. a) Evaporation performance of the lab‐scale iSTPV at a high evaporation rate for 32 h. Inset, photographs showing the solute accumulation process. b) Photograph of the iSTPV used for outdoor test on the lawn at Nanjing University Xian Lin campus. Scale bar, 3.5 cm. c) Solar intensity, ambient temperature, and relative humidity of two sunny days during the evaluation of the evaporation performance of the iSTPV before and after solute accumulation over time from 6:00 a.m. to 6:00 p.m. d) Photograph of the iSTPV's surface before solute accumulation showing the 24 evaporation unit with the overall evaporation area of 15 cm × 25 cm. Scale bar, 3.5 cm. e) Photograph of the iSTPV showing solute (Ni^2+^) accumulation after water evaporation. Scale bar, 3.5 cm. f) The mass loss of water for the iSTPV before and after solute accumulation on the surface of the air‐laid paper during the outdoor test. g) PH of the solution before and after purification. h) Water purification performance of the iSTPV compared with several competitive purification techniques.

The long‐term stability of iSTPV at higher evaporation rates was also proved. The distance between the emitter and paper was increased to 15 mm with the evaporation area decreased to 2 cm × 2 cm so as to simulate the situation of large area application.^[^ ^14b^ ^]^ As can be seen from **Figure** **4**a and Video S1 in the Supporting Information, the iSTPV maintained a high evaporation rate of over 1.94 kg m^−2^ h^−1^ under 2.6‐sun illumination in the 32 h test. And the solute layer can be removed easily without damaging the underlying paper layer (Figure S6, Supporting Information). We also built a larger iSTPV with an evaporation area of 15 cm × 25 cm, which consists of 24 evaporation units, and evaluated its outdoor performance (Figure 4b). As can be seen from Figure 4f, the total mass change of water before and after solute accumulation (Figure 3d,e) shows little difference in two similar sunny days (Figure 4c), demonstrating its stability.

Last, the vapor quality was carefully examined. Saturated Ni^2+^ and Cu^2+^ solutions and 20 wt% Zn^2+^ and Cd^2+^ solutions were used for the evaluation of water purification performance of iSTPV. PH test of the solution before and after purification showing that the purified water is neutral. Inductively coupled plasma‐mass spectrometry tests show that the concentrations of heavy metal ions dropped below 0.5 mg L^−1^ after purification, and the purification capacity are comparable with those of competitive purification techniques designed for specific ion.^[^ ^18^ ^]^

## 3 Conclusion

Sustainable solar evaporation in presence of solute accumulation was achieved via an interfacial solar thermal photo‐vapor generator. The prototype evaporator can evaporate at a high speed of 1.94 kg m^−2^ h^−1^ during a persistent solute accumulation process for 32 h. This finding provides an exciting path to promote advanced solar evaporators so as to ease global water crisis.

## 4 Experimental Section

##### Materials Preparation

The solar selective absorber was provided by Almeco (absorptance: 0.92 and emittance: 0.04). Black paint emitter (4SD Hi‐Temp Paint) was sprayed on the back of the SSA for 2 even layers. Bubble wrap with a transmittance of 0.86 was used as the convective cover. Air‐laid paper was purchased from Kimwipes (KIMTECH, US). For the fabrication of iSTV, 6 mL TiN nanofluids (3 mg L^−1^) was sprayed on the air‐laid paper (45 mm × 45 mm).

##### Materials Characterizations

The SEM images were captured by EM30 (COXEM, Korea). Optical contact angle meter 30 (Dataphysics Instruments Gmbh, Germany) was used to evaluated the water permeate speed of the air‐laid paper. Infrared photos were captured by UTi160b (Uni‐Trend Technology, China).

##### Vapor Generation and Waste Water Treatment

The MIR source was produced by an infrared lamp (PAR38, PHILIPS, Poland). CEL‐S500 with an AM 1.5G filter was used to simulated the solar illumination in the lab. The ambient conditions were recorded by a hygrometer (Oneset, HOBO UX100). PR224ZH/E (0.1 mg accuracy) was used to record the mass change of water. NiSO_4_·(NH_4_)_2_·SO_4_·6H_2_O, CuSO_4_·5H_2_O, Zn(CH_3_COO)_2_, and Cd_2_·5H_2_O were used as model metal ions in wastewater. The ambient temperature of lab was kept 30 °C with humidity of ≈40% during the vapor generation process.

## Conflict of Interest

The authors declare no conflict of interest.

## Supporting information

Supporting Information

Supplemental Video 1
