# Supplementary material for: Sustainable Solar Evaporation from Solute Surface via Energy Downconversion
Source: Glob Chall. 2020 Oct 26;5(1):2000077. doi: 10.1002/gch2.202000077 (PMC7788599; doi:10.1002/gch2.202000077)
Supplement: Supplementary file 1 — Supporting Information [file GCH2-5-2000077-s001.pdf]

© 2020 Wiley-VCH GmbH

Global Challenges

Supporting Information

for *Global Challenges,* DOI: 10.1002/gch2.202000077

Sustainable Solar Evaporation from Solute Surface via Energy Downconversion

Yue Bian, Yanli Tian, Kun Tang, Wei Li, Lijuan Zhao, Yi Yang, Jiandong Ye, and Shulin Gu**

Copyright Wiley-VCH GmbH, Germany, 2020.

Supporting Information

Sustainable Solar Evaporation from Solute Surface via Energy Down-conversion

Yue Bian, Yanli Tian, Kun Tang, Wei Li, Lijuan Zhao, Yi Yang, Jiandong Ye and Shulin Gu**

Supporting Figures

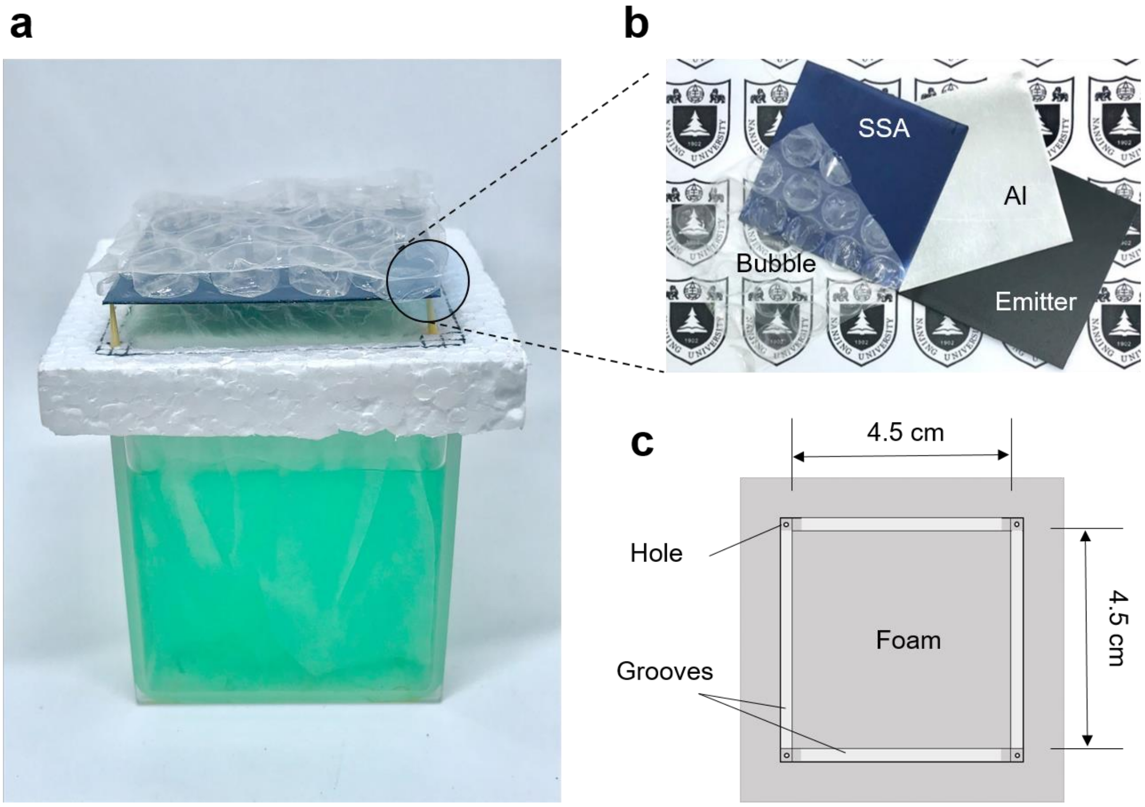

Figure S1. a) Picture of the iSTPV. b) Photos of the bubble wrap, solar selective absorber (SSA), back side of the SSA showing the aluminum substrate, Al substrate after painted with the black emitter. c) Sketch map shows that four grooves (width: 2mm) are excavated from the foam, creating an evaporation area of 45mm×45mm after putting into the paper layer. Toothpicks are insert into the four holes in the cornors so as to suppot the bubble and SSA. The distance between the SSA and paper layer is 8 mm.

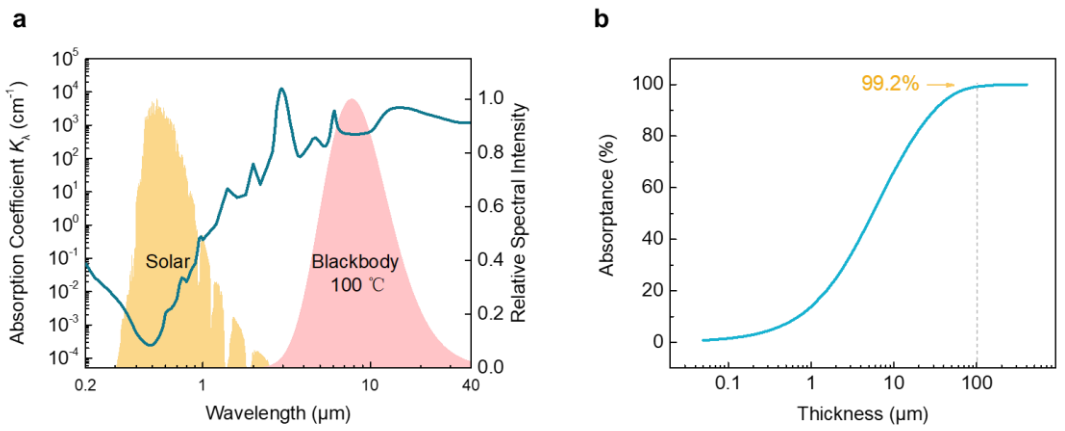

Figure S2. a) Absorption coefficient of water showing the low absorptance in the solar spectral range and high absorptance in the MIR-FIR spectrum range. b) Calculated absorptance of a thin water layer with different thickness (weighted by the 100 ℃ blackbody emitter).

The absorption of a beam of radiation as it propagates through a medium can be calculated by the Beer-Lambert law

$$A_\lambda(L) = 1 - I_\lambda(L)/I_{\lambda,0} = 1 - e^{-\beta_\lambda L} \quad (1)$$

where $A_\lambda(L)$ is the spectral absorptance, defined as the 1 minus the intensity I_λ of a beam at a distance L, relative to the incident intensity $I_{\lambda,0}$ at L=0. And β_λ is the spectral extinction coefficient. In the absence of scattering, the extinction coefficient is equal to the absorption coefficient K_λ, which can be found from the imaginary part k of the complex refractive index $\tilde{n}=n-ik$

$$K_\lambda = 4\pi k(\lambda)\lambda^{-1} \quad (2)$$

It is apparent that water absorbs light strongly in the MIR-FIR range rather than the solar spectrum range (Figure S2a). When considering broadband radiation, e.g. solar or blackbody radiation, it is necessary to spectrally average the absorption coefficient. The internal absorptance at a given depth into water is calculated from

$$A_{int}(L) = 1 - \frac{I(L)}{I_0} = 1 - \frac{\int_0^{\infty} I_\lambda(L) d\lambda}{\int_0^{\infty} I_{\lambda,0} d\lambda} = 1 - \frac{\int_0^{\infty} I_{\lambda,0} e^{-K_\lambda L} d\lambda}{I_0} \quad (3)$$

Figure S2b shows the calculated absorptance of a thin water layer with different thickness in the MIR-FIR range. As can be seen, the water layer of 100 μm can readily absorb 99.2% of the infrared photons. Considering that typical blackbody radiation is a diffuse beam, the required absorption depths for a thermal source tend to be lower than 100 μm.[1].

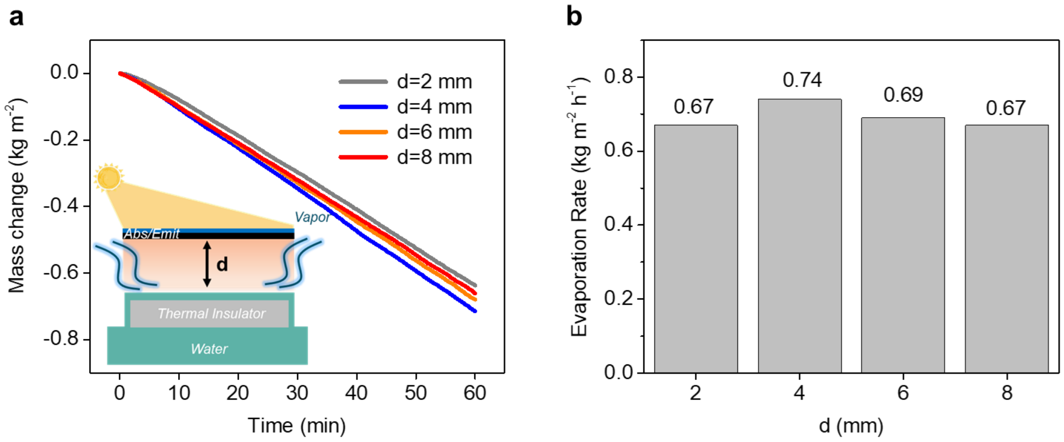

Figure S3. Effects of the air gap distance between the evaporation front and emitter layer. a, b) Mass changes of pure water over time a) and solar vapor generation rate b) under 1-sun illumination using iSTPV.

When we refer to similar energy down-conversion design without 2D water supply,[2] the distance between the absorber and water surface was set ~4 mm. We note that the air gap between the evaporation layer and the blackbody emitter was set 8 mm in our manuscript, which was reasonably designed for the lab-scale evaporator. The distance between the evaporation layer and the emitter layer does affect the evaporation rate as a high distance owns a low emittance view factor while a low distance may be an obstacle to vapor escape. Therefore, in order to study the evaporation performance in the process of solute deposition, the impact from the distance change itself should be minimized. Actually, when designing the initial distance between the absorber and evaporation surface, we first compared the

evaporation rate of iSTPVs with four different distances under 1-sun (Figure S3a). As can be seen from Figure S3b, the highest evaporation rate occurs at the air gap distance of 4 mm. Further increasing the distance, the evaporation rate showed a downward trend, which could be attributed to the decreased emitter view factor with increasing air gap. A diffusion-limited mode occurs when the air gap is very small (d=2mm). Note that there is no significant difference in the evaporation rate of iSTPV when the air gap is 6 mm and 8mm. So the initial air gap was set 8 mm in the manuscript.

We note that the spacing can be enlarged in proportion to the size of the evaporation pond when used in practical applications. For example, 98% the radiation leaving the emitter can be intercepted by the evaporation surface in an evaporation pond with side length of 10 m and air gap of 10 cm. So the distance between the emitter and evaporation surface could be very large which would be of no problem for vapor escape.

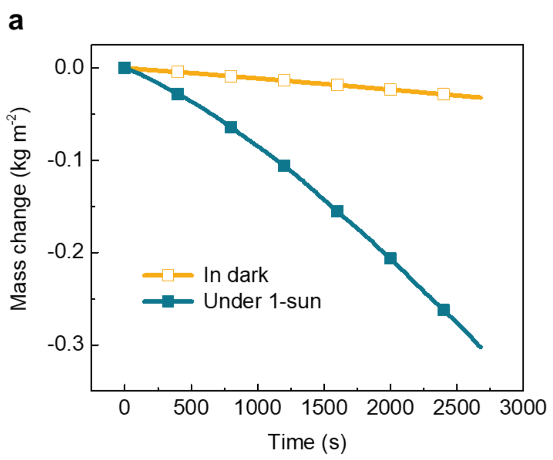

Figure S4. Mass change (a) and evaporation rate (b) of bare Ni^{2+} solution in dark and under 1-sun illumination.

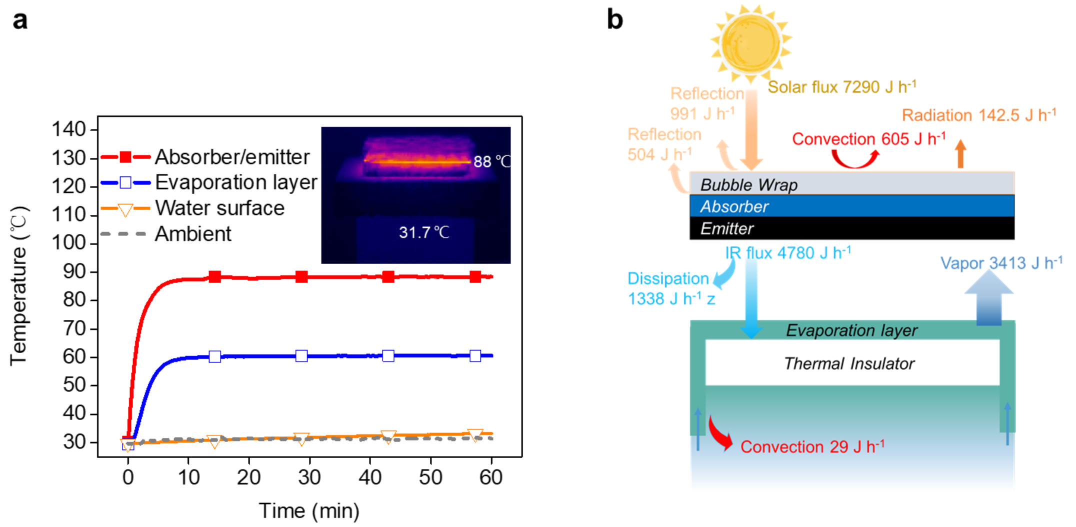

Figure S5. Heat behavior of the interfacial solar thermal photo vapor-generator (iSTPV). a) Temperature evolution of the iSTPV. Inset, infrared image of the iSTPV after illumination for 1h. b) Schematic of the heat behavior in the iSTPV.

The temperature evolution of the iSTPV under 1-sun illumination was investigated. As shown in Figure 5a, the temperature of the absorber increases rapidly to a steady-state temperature of 88 ℃ in less than 7 min and the thin water layer reaches a steady temperature of 60.5 ℃ almost at the same time as the absorber/emitter. The downward thermal loss from the hot paper layer to bulk water is suppressed by using the 2D water supply design, which is intuitive from the infrared image (insert in Figure S5a) - although the temperature of the evaporation surface is as high as 60.5 ℃, the surface temperature of the bottom water only rises by 1.7 ℃ after 1-h illumination.

Figure S5b shows the schematic of the heat behavior in the iSTPV. The overall system efficiency (η) is:

$$\eta = \dot{m} h_{LV} / q_{solar} = \eta_1 \times \eta_2 \times \eta_3 \qquad (4)$$

where $\dot{m}$ is the net evaporation rate (with dark evaporation subtracted) , h_{LV} is the vaporization heat of water (i.e., ≈2349 J g^{-1} for pure water at 60.5 ℃[3]), q_{solar} is the solar flux (i.e., 1 kW m^{-2} for 1-sun at AM 1.5). There are three parts to this efficiency: η_1 represents the bubble/absorber optical efficiency, η_2 incorporates the emitter efficiency as well as the radiative coupling between the solar umbrella and water surface, and η_3 represents the fraction of incident radiation on the water surface that is used for evaporation.

The bubble/absorber optical efficiency (η_1) can be calculated by:

$$\eta_1 = \frac{t_{bubb} a_{abs} A_{abs} q_{solar} - A_{abs} \varepsilon_{abs} \sigma (T_{abs}^4 - T_{env}^4) - A_{air-bubb} h \ (T_{abs} - T_{env})}{A_{abs} q_{solar}}$$

(5)

where t_{bubb} is the transmittance of the bubble wrap (0.86), a_{abs} is the absorptance of the solar selective absorber (SSA, 0.92), ε_{abs} is the emittance of the SSA (0.04), A_{abs} is the projection area of the SSA, $A_{air-bubb}$ is the air area of the bubble wrap (~0.285A_{abs}), σ is the Stefan-Boltzmann constant (i.e., 5.67×10^{-8} W m^{-2} K^{-4}), h is the convection heat transfer

coefficient (5 W m^{-2} K^{-1}), T_{abs} is the temperature of the SSA, and T_{env} is the temperature of the environment. Supplementary Table 1 summarizes the energy input and loss channels of the iSTPV in the solar light absorption process.

Supplementary Table 1. Summary of energy gain and loss channels of the iSTPV in the solar light absorption process.

Light input (J h^{-1})	Reflection loss (J h^{-1})		Convection (J h^{-1})	Radiation (J h^{-1})	Net energy input (J h^{-1})
7290	991 (Bubble)	504 (SSA)	605	142.5	5047.5

It can be inferred from Supplementary Table 1 that η_1=5047.5/7290=69.2%.

And, η_2=Fε_{emit}=68%, where F is the view factor of the emitter in our iSTPV (0.72),[2] ε_{emit} is the the emittance of the blackbody emitter (94.7%). The energy loss from the hot paper layer to the bulk water can be calculated by Q=Cm△T/t, where C is the specific heat capacity of water (4.2×10^3 J K^{-1} kg^{-1}), m is the mass of the bottom bulk water (~4.05×10^{-3} kg), △T is the temperature increase of water (~1.7 K in 1 h). About 29 J h^{-1} energy was lost to the bulk water, leaving ~3413 J h^{-1}for evaporation. Thus, according to the steady-state energy balance analysis the total energy efficiency (η) should be around η=3413/7290=46.8%. The experimental energy efficiency is $\eta=\dot{m}h_{LV}/q_{solar}$=0.67 kg m^{-2} h^{-1}×2349 J g^{-1}/1 kW m^{-2}=43.7%, which matches well with the theoretical value.

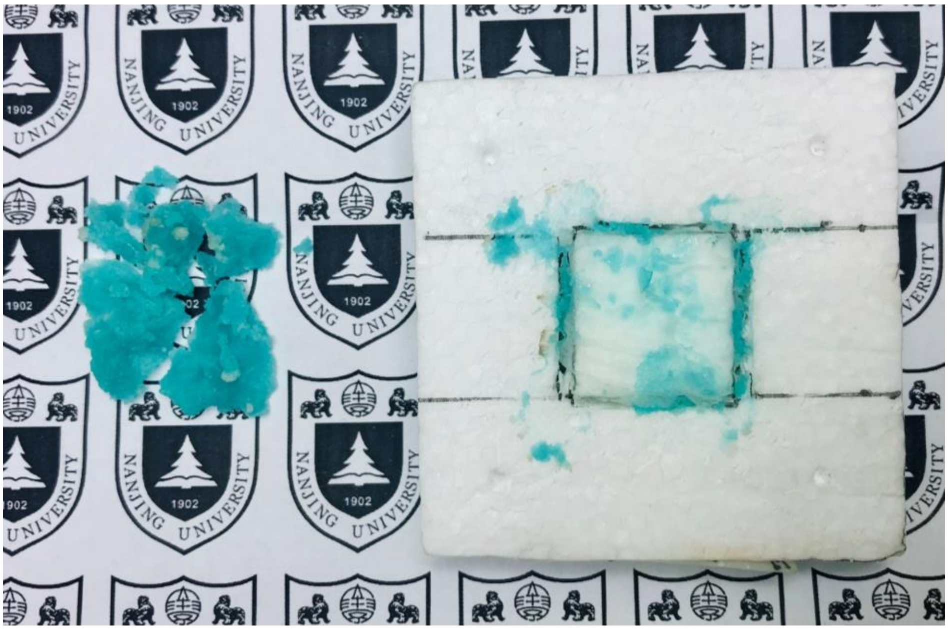

Figure S6. Photograph of the iSTPV after solute removal.

Supporting Video

Video S1. Evaporation performance of the lab-scale iSTPV at a high evaporation rate for 32 h.

Supplementary References

[1] T. A. Cooper, S. H. Zandavi, G. W. Ni, Y. Tsurimaki, Y. Huang, S. V. Boriskina, G. Chen, *Nat. Commun.* **2018**, 9, 5086.

[2] A. K. Menon, I. Haechler, S. Kaur, S. Lubner, R. S. Prasher, *Nat. Sustain.***2020**, 3, 144.
